# Postpartum Acute Liver Dysfunction: A Case of Acute Fatty Liver of Pregnancy Developing Massive Intrahepatic Calcification

**DOI:** 10.14740/gr693w

**Published:** 2015-12-31

**Authors:** Khalid Javid Bhat, Rabia Shovkat, Hamad Jeelani Samoon

**Affiliations:** a Department of Internal Medicine, GMC & Hospitals, Srinagar, Kashmir 190010, India; b Department of Internal Medicine, ASCOMS & Hospitals, Sidhra, Jammu, Jammu & Kashmir, 180017, India; c Department of Internal Medicine, Safdarjang Hospital, Ansari Nagar, New Delhi 110029, India

**Keywords:** Acute fatty liver of pregnancy, Massive intrahepatic calcification, Acute kidney injury

## Abstract

The function of the liver is particularly affected by the unique physiologic milieu of the pregnancy. Pregnancy-related liver diseases encompass a spectrum of different etiologies that are related to gestation or one of its complications. Hepatic calcification, a rare entity, is usually associated with infectious, vascular, or neoplastic lesions in the liver. To the best of our knowledge, only one case of rapidly occurring pregnancy-related intrahepatic calcification has been documented in a patient with severe eclampsia or hemolysis, elevated liver enzymes and low platelet count (HELLP) syndrome. Here we present a case of immediate “postpartum” acute fatty liver of pregnancy (AFLP) in a 23-year-old hypertensive primigravida, complicated by acute renal dysfunction who developed dense intrahepatic calcification in less than a month after the initial diagnosis. A multidisciplinary approach for the management was used, to which the patient responded aptly. This case illustrates the first description of intrahepatic calcification in AFLP syndrome and highlights some of the challenges met in making the final diagnosis.

## Introduction

Acute fatty liver of pregnancy (AFLP) is a rare life-threatening complication with incidence of one in 10,000 to 15,000 pregnancies and a mortality rate of approximately 18% [1]. It usually occurs in the third trimester of pregnancy and as late as the immediate postpartum period [2]. It presents with features ranging from progressive jaundice to fulminant liver failure. In most of the patients who recover, there is no permanent or long-term sequela of the liver disease itself.

AFLP as a cause of intrahepatic calcification is seldom known. A wide array of causes have been described worldwide, and the most common causes are calcified granulomas and hydatid cyst, followed by calcification associated with hepatic neoplasms [3]. Hepatic calcifications which are dense and diffuse are rare and have multifactorial causation, sometimes unrelated to the liver diseases. We present a case of immediate postpartum AFLP complicated by acute kidney injury (AKI), in whom the intrahepatic calcifications appeared rapidly, 4 weeks after the initial diagnosis. A brief literature review and the pathophysiology of the hepatic calcification and renal failure in AFLP is discussed.

## Case Report

**Figure 1:**
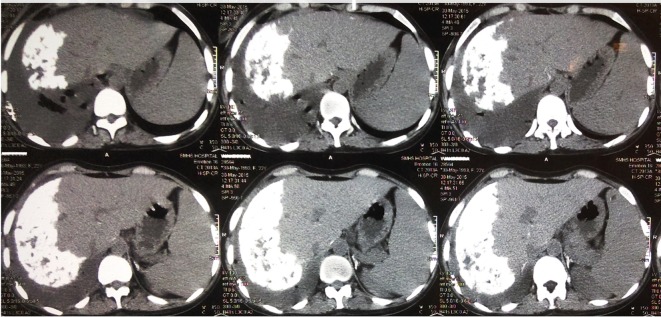
CT abdomen, axial view with serial images showing dense calcification in right hepatic lobe with pleural effusion.

**Figure 2:**
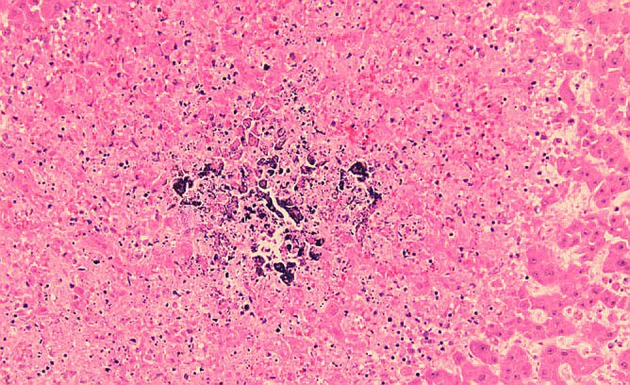
Liver biopsy showing calcified deposits with mild foamy reaction in the background.

In April 2015, a 23-year-old primigravida of Indian origin underwent a lower segment cesarean section at a community hospital and had delivered a healthy male baby following which she was referred to this center with the complaints of jaundice, pain upper abdomen and decreased urine output for 1 day. The abdominal pain was of sudden onset, moderate in severity associated with few episodes of vomiting. One episode of severe hypoglycemia (26 mg/dL) was documented prior to the referral and she had received two fresh blood transfusions for excessive uterine bleeding. She was hypertensive for last 2 years and was on oral labetalol 100 mg and methyldopa 250 mg twice daily. On examination, she appeared drowsy, and had blood pressure of 110/80 mm Hg, pulse of 112 beats/min and respiratory rate of 24 breaths/min. Abdominal examination revealed a mild upper quadrant tenderness and a healthy surgical wound. Investigations revealed hemoglobin of 7.6 g/dL, total leucocyte count of 32,600/mm^3^, predominantly neutrocytic with platelet count of 34,000/mm^3^. Peripheral smear showed normocytic normochromic anemia. Her random blood sugar was 33 mg/dL and she was immediately started on 10% dextrose infusion. Serum levels of bilirubin, aspartate transaminase (AST), alanine transaminase (ALT) and amylase were 3.9 mg/dL, 169 U/L, 346 IU/L, and 166 IU/L respectively. Coagulogram revealed a prothrombin time (PT) of 22 s with international normalized ratio (INR) of 2.5. Hepatitis serology, autoimmune markers and dengue infection screening were negative. There were moderately increased blood levels of urea (125 mg/dL) and creatinine (2.29 mg/dL) and a pronounced increase of serum uric acid 9.6 mg/dL. Serum electrolytes showed potassium of 5.2 mEq/L, serum sodium of 133 mEq/L and serum calcium of 9.9 mg/dL. Abdominal ultrasonography (USG) revealed an enlarged liver with segmental edema, mild ascites and right-sided pleural effusion. She was shifted to intensive care unit in view of the altered liver function and impending renal failure. On seventh postpartum day, her hemoglobin was 4.5 mg/dL, serum urea of 279 mg/dL, serum creatinine of 7.23 mg/dL, serum uric acid of 12 mg/dL, serum bilirubin of 24 mg/dL (predominantly conjugated), AST of 177 IU/L, ALT of 447 IU/L, LDH of 447 IU/L and INR of 2.9. Blood and urine cultures were sterile and funduscopic examination was normal. Urine output was 400 mL/24 h and she was put on dialysis protocol for AKI. A working diagnosis of acute liver failure was made and she was closely monitored. Over the next 2 weeks, she required several sessions of intermittent dialysis, eight units of blood transfusions, and 18 units of fresh frozen plasma/cryoprecipitate to maintain an INR of < 1.5. Meanwhile there was a gradual improvement in her general status, normalization of aminotransferase activity and renal function. Subsequently 2 weeks later, in May 2015, abdominal USG was repeated which showed bright heterogeneous echogenicity of the right lobe of the liver. A non-contrast abdominal computed tomography showed intrahepatic calcifications predominant in the right lobe of the liver and right-sided pleural effusion. The greatest densities were located in the segments 6, 7 and 8 of the liver (Fig. 1). Hydatid cyst serology and alpha-feto-protein assay were insignificant. She was discharged with complete clinical and biochemical recovery in June 2015. A follow-up abdominal USG performed 2 weeks later showed stable intrahepatic calcifications and a USG-guided biopsy, to rule out any rare malignant cause, showed punctuated calcifications with a foamy residual cell infiltrate (Fig. 2).

## Discussion

Acute postpartum liver dysfunction can be due to traditional pregnancy-related liver disorders itself or a part of sepsis syndrome. Liver disease in pregnancy encompasses a spectrum of different etiologies with both severe pre-eclampsia/HELLP syndrome and AFLP recognized as multi-organ dysfunctions that are unique to pregnancy. Their clinical presentation and laboratory findings may overlap, allowing a potential delay in the diagnosis. AFLP is more common in first and multiple pregnancies and a mitochondrial enzyme deficiency, long chain 3-hydroxyacetyl coenzyme-A dehydrogenase (LCHAD) is frequently encountered [4]. The initial working diagnosis in our patient posed a diagnostic challenge and we had a difficulty in differentiating AFLP from severe eclampsia/HELLP, and hemolytic uremic syndrome (HUS)/thrombotic thrombocytopenic purpura (TTP). In general, postpartum HELLP syndrome amounts to about 30% of cases while as AFLP is very rare in postpartum period [5]. The diagnosis of AFLP in our patient was assisted by the Swansea criteria which needs minimum six of the following criteria: vomiting, abdominal pain, azotemia, polydipsia/polyuria, encephalopathy, ascites, hyperbilirubinemia, hypoglycemia, hyperuricemia, hyperammonemia, leucocytosis, transaminites, coagulopathy, bright echotexure on ultrasound scan and/or microsteatosis on liver biopsy [6]. Noteworthy, hypertension is present in 30-50% cases of AFLP, but its invariably presents in HELLP/severe eclampsia [7]. Although our patient was hypertensive, we did not find any evidence of hemolysis or proteinuria in our patient, suggestive of severe eclampsia/HELLP syndrome. The recurrent hypoglycemia, clouded consciousness, predominantly conjugated serum bilirubin with mild elevation of transaminase levels were the key features of AFLP which distinguished it from HELLP syndrome in our patient [8]. In addition some laboratory investigations can be adjunctive. Prolongation of PT, INR and elevated creatinine concentration is more common in AFLP than with HELLP syndrome. Our patient developed anemia due to acute blood loss and not due to any hemolytic or microangiopatic syndromes like HUS, since overt reticulocytosis, red cell fragmentation, markedly elevated LDH levels or indirect bilirubin were absent in our case. A liver biopsy may facilitate the definite distinction between AFLP and HELLP syndrome, but owing to concerns regarding fulminating coagulopathy, it is generally avoided [9]. Similarly in the absence of isolated platelet consumption and elevated LDH, TTP-HUS was ruled out. One significant finding in our patient was the development of AKI. The incidence of AKI in AFLP is about 60% in the US, but the pathogenesis of AKI in AFLP during pregnancy is not well understood [10]. Hyperuricemia was initially disproportionately high in comparison with renal dysfunction, a characteristic of AFLP.

Our case represents the first description of intrahepatic calcification with the waning of AFLP syndrome in less than a month after childbirth. Common causes of intrahepatic calcification include infections (tuberculosis, hydatid cyst, schistosomiasis, toxoplasmosis, chronic amebic or pyogenic abscess), benign lesions (hemangioma, adenoma), hepato-cellular carcinoma and metastatic tumors of the liver [3]. In our patient, the calcification was dense and deep seated and present predominantly in the right lobe of the liver. Only one case of pregnancy-related intrahepatic calcification attributed to severe eclampsia or HELLP syndrome that appeared 3 weeks after the initial diagnosis is documented [11]. The precise mechanism of hepatic calciphylaxis is not clear. Parenchymal liponecrosis appearing synergistically with renal dysfunction seems to be a plausible explanation. Indeed renal failure is the most important risk factor associated with the development of systemic calciphylaxis and diffuse intrahepatic calcifications are reported in patients on hemodialysis with/or without noticeable hyperparathyroidism [12]. Abnormal intracellular calcium homeostasis or elevated calcium-phosphorus products may be the underlying mechanism in renal insufficiency. Alternate mechanism includes ischemic liver injury from shock or hypotension which provokes smooth muscle cell de-differentiation to osteoblast-like cells and calciphylaxis via nuclear factor κB (NFκB) activation [13].

In conclusion, the AFLP syndrome should be recognized as a possible cause of intrahepatic calcifications and early diagnosis and adequate supportive care are the key to good outcome of this unique pregnancy-related liver dysfunction.
